# Opioid receptor signaling, analgesic and side effects induced by a computationally designed pH-dependent agonist

**DOI:** 10.1038/s41598-018-27313-4

**Published:** 2018-06-12

**Authors:** Viola Spahn, Giovanna Del Vecchio, Antonio Rodriguez-Gaztelumendi, Julia Temp, Dominika Labuz, Michael Kloner, Marco Reidelbach, Halina Machelska, Marcus Weber, Christoph Stein

**Affiliations:** 1 Charité - Universitätsmedizin Berlin, corporate member of Freie Universität Berlin, Humboldt-Universität zu Berlin, and Berlin Institute of Health, Department of Anesthesiology and Intensive Care Medicine, Campus Benjamin Franklin, Hindenburgdamm 30, 12203 Berlin, Germany; 2 Freie Universität Berlin, Institute of Theoretical Physics, Arnimallee 14, 14195 Berlin, Germany; 3 Zuse Institute Berlin, Computational Molecular Design, Takustraße 7, 14195 Berlin, Germany; 4 Present Address: Department of Drug Discovery and In Vitro Pharmacology, Laboratorios Dr. Esteve, Parc Científic de Barcelona, Barcelona, Spain

## Abstract

Novel pain killers without adverse effects are urgently needed. Opioids induce central and intestinal side effects such as respiratory depression, sedation, addiction, and constipation. We have recently shown that a newly designed agonist with a reduced acid dissociation constant (pK_a_) abolished pain by selectively activating peripheral μ-opioid receptors (MOR) in inflamed (acidic) tissues without eliciting side effects. Here, we extended this concept in that pK_a_ reduction to 7.22 was achieved by placing a fluorine atom at the ethylidene bridge in the parental molecule fentanyl. The new compound (FF3) showed pH-sensitive MOR affinity, [^35^S]-GTPγS binding, and G protein dissociation by fluorescence resonance energy transfer. It produced injury-restricted analgesia in rat models of inflammatory, postoperative, abdominal, and neuropathic pain. At high dosages, FF3 induced sedation, motor disturbance, reward, constipation, and respiratory depression. These results support our hypothesis that a ligand’s pK_a_ should be close to the pH of injured tissue to obtain analgesia without side effects.

## Introduction

Opioid receptor agonists are the most powerful drugs to treat severe acute and cancer-related pain. However, major problems have emerged due to their epidemic misuse and adverse effects^1^. These side effects comprise sedation, respiratory depression, addiction, nausea, and constipation, and are mediated by central or intestinal opioid receptors^2,3^. The analgesic effects result from the activation of both central and peripheral opioid receptors *via* inhibitory G-proteins, which dissociate into G_αi_ and G_βγ_ subunits. Further downstream signaling leads to suppression of adenylyl cyclases and the modulation of ion channels, resulting in an overall decrease in neuronal excitability^2^. Many experimental and clinical studies revealed that a substantial proportion of opioid analgesia is mediated by the activation of opioid receptors on peripheral sensory neurons (reviewed in^3,4^). Numerous pain syndromes (e.g. arthritis, neuropathy, postoperative pain, cancer) are accompanied by injury-induced tissue acidosis and upregulation of such peripheral opioid receptors^3,5–7^. Therefore, the potential of peripheral opioid receptors as drug targets is increasingly recognized^8,9^. In contrast to previous pharmacokinetic concepts (reviewed in^3^), we recently developed a new pharmacodynamics-based design for peripherally-acting opioids lacking central or intestinal side effects^5^. This strategy is based on computational simulations of pathological receptor conformations and the finding that the protonation state of a ligand is crucial for its activity at opioid receptors. We hypothesized that the ligand’s pK_a_ should be reduced to values close to the acidic pH of injured tissue. This was achieved by fluorination of the piperidine ring in the μ-opioid receptor (MOR) agonist fentanyl, leading to the compound (±)-*N*-(3-fluoro-1-phenethylpiperidine-4-yl)-*N*-phenyl propionamide (NFEPP). In addition, our original computer simulations suggested hydrogen/fluorine exchange in fentanyl′s ethylidene bridge^5^. Accordingly, the compound (±)-*N*-[1-(2-fluoro-2-phenylethyl)piperidine-4-yl]-*N*-phenyl propionamide (FF3) was synthesized by a contractor (ASCA GmbH Berlin) and tested *in vitro* and *in vivo* in the present study.

## Results

### Computational design of FF3

**Figure 1:**
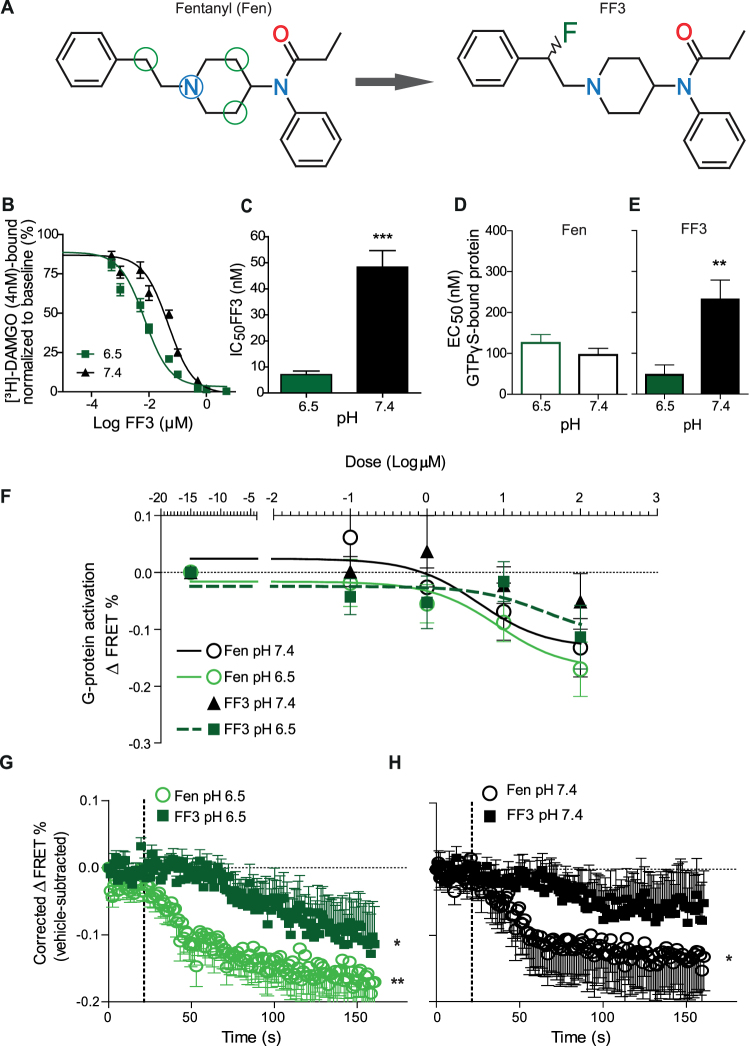
FF3 preferentially binds to and activates MOR at low pH in HEK293 cells. (**A**) Chemical structures of fentanyl (Fen) (left) and (±)-*N*-[1-(2-fluoro-2-phenylethyl)piperidine-4-yl]-*N*-phenyl propionamide (FF3) (right). The blue circle highlights the acidic nitrogen atom subjected to pH-dependent protonation in whose vicinity electrons may be withdrawn to reduce the pK_a_ value. Green circles denote CH_2_-groups where a single hydrogen may be replaced by a fluorine. (**B**) Displacement of bound [³H]-DAMGO by FF3 at pH 6.5 and 7.4. (**C**) IC_50_ calculated from B) at pH 6.5 and 7.4 (***P < 0.001, unpaired t-test, n = 6). (**D**,**E**) EC_50_ of fentanyl- (**D**) and FF3- (**E**) induced [^35^S]-GTPγS-binding at pH 6.5 and 7.4 (**P < 0.01, unpaired t-test, n = 5–6). (**F**) Gα_i_-mediated FRET responses (ΔFRET between Gα_i_-mTqΔ6 and cpVenus-Gγ_2_) to fentanyl and FF3. FF3 at pH 7.4 did not induce significant ΔFRET % compared to vehicle. Curves are derived by nonlinear regression fits constrained to each maximum effect. (**G**) Time course of FRET responses to fentanyl and FF3 (100 μM) at pH 6.5 differ from those of vehicle-treated cells (*P < 0.05, **P < 0.01 vs. vehicle-treated cells at the corresponding pH, one-way RM-ANOVA and Dunnett’s test, n = 10). (**H**) At pH 7.4, only fentanyl (100 µM) induced significant FRET responses compared to vehicle (*P < 0.05 vs. vehicle-treated cells at the corresponding pH, one-way RM-ANOVA and Dunnett’s test, n = 10). Data are means ± SEM.

Our previous *in silico* studies demonstrated that exchanging hydrogen by fluorine at distinct positions in the fentanyl molecule decreases its pK_a_ value^5^. An electron withdrawing moiety within a distance of two carbon bonds (Fig. 1A, green circles) of the acidic nitrogen atom (Fig. 1A, blue circle) is able to decrease the pK_a_ such that the new molecule (FF3) is protonated in acidic and deprotonated in healthy tissue^5^. By choosing the fluorine atom, one hydrogen is replaced by only one other atom. Therefore, the overall size, geometry, and MOR binding properties of FF3 should not change significantly. To estimate its pK_a_, we used the B3LYP functional with a 6–31 + G* basis. In contrast to single starting structures used previously^5^, ensembles of structures derived from molecular dynamics simulations were now the basis for the calculations yielding an averaged pK_a_ value of 6.01. Experimental measurements by a contractor resulted in a pK_a_ of 7.22 (Table 1).

**Table 1:** pK_a_, IC_50_ and EC_50_ values of FF3, fentanyl and NFEPP.

Molecule	**FF3**		**Fentanyl**	**NFEPP**	
Structure					
Replaced hydrogen (indexed above and in^5^)	12	13	—	3	7
Calculated pKa	6.31 ± 1.18	5.71 ± 0.89	9.11 ± 0.14	6.73	6.93
	Mean = 6.01			Mean = 6.83	
Function for pK_a_ calculation	B3LYP/6-31 + G*//B3LYP/6-31 + G*			B3LYP/6-31 + G*//HF/6-31 + G* published in^5^	
Experimentally obtained pK_a_ at 25 °C	7.22 ± 0.01^+^		8.43 ± 0.05^32,33^	6.82 ± 0.06^5^	
IC_50_ (nM) (Binding)					
pH 6.5	7.1 ± 1.4		6.9 ± 1.1^#^	18.3 ± 3.3^#^	
pH 7.4	48.3 ± 6.4		4.8 ± 0.7^#^	81.9 ± 22.1^#^	
EC_50_ (nM) (GTPγS)					
pH 6.5	47.2 ± 24.2		125.5 ± 20.6	Not determined	
pH 7.4	231.4 ± 47.9		96.1 ± 16.6	Not determined	
EC_50_ (nM) (FRET)					
pH 6.5	33.92		8.31	Not determined	
pH 7.4	Not obtainable		4.74	Not determined	

^+^This pK_a_ value was experimentally measured by a contractor (Sirius Analytical, UK).

^#^These IC_50_ values were the basis for the K_i_ value calculation published in^5^.

### FF3 preferentially binds to and activates MOR at low pH

In membrane preparations of MOR-transfected Human Embryonic Kidney-293 (HEK293) cells, FF3 showed increased potency to displace the radioactively-labeled standard MOR ligand [^3^H]-[D-Ala^2^, N-MePhe^4^, Gly-ol]-enkephalin (DAMGO) (4 nM), as demonstrated by the significantly reduced IC_50_ at pH 6.5 compared to physiological pH 7.4 (Fig. 1B,C). In the [^35^S]-GTPγS binding assay, the EC_50_ of FF3 was significantly lower at pH 6.5 than at pH 7.4 (Fig. 1E). In contrast, we found no pH-dependent differences in fentanyl-induced G-protein activation in this assay (Fig. 1D). Using real-time fluorescence resonance energy transfer (FRET) in HEK293 cells transfected with MOR, Gβ_1_, Gα_i1_-mTqΔ6, and cpVenus-Gγ_2_, we observed dose-dependent dissociation of G-protein subunits (ΔFRET between Gα_i1_-mTqΔ6 and cpVenus-Gγ_2_) upon treatment with fentanyl at both pH 7.4 and 6.5, while FF3 induced dose-dependent effects only at pH 6.5 but not at pH 7.4 (no fitting was found, Fig. 1F). At pH 6.5 (Fig. 1G), time courses of FRET responses to both fentanyl and FF3 (100 μM) differed significantly from those of vehicle-treated cells, whereas at pH 7.4 only fentanyl induced significant effects compared to vehicle (Fig. 1H).

### FF3 produces analgesia selectively in injured tissue

**Figure 2:**
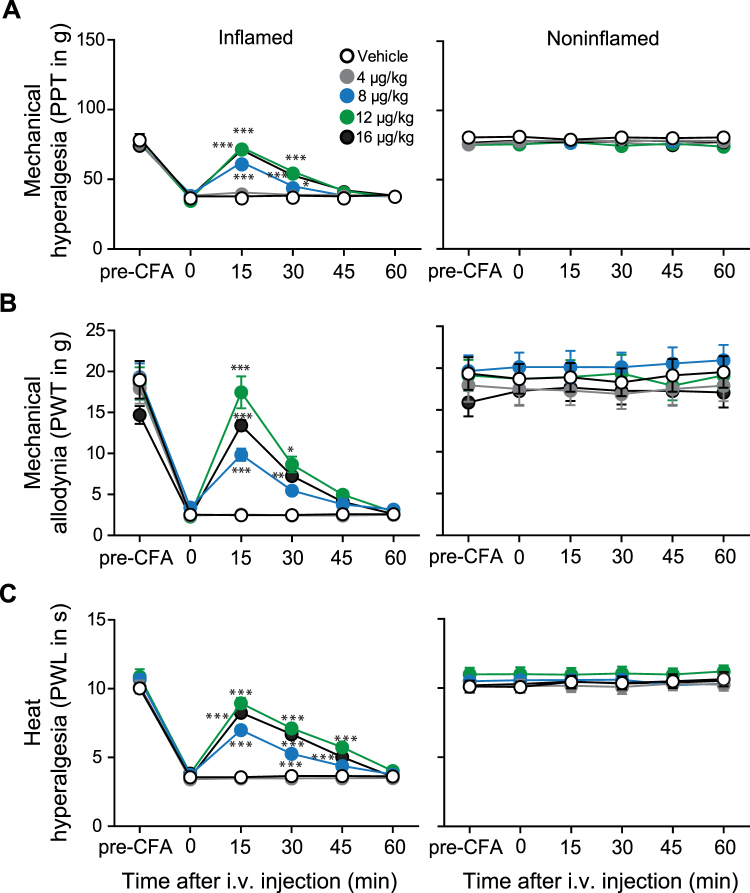
Systemic FF3 dose-dependently reduces pain selectively in inflamed tissue in the CFA model. Effects after intravenous (i.v.) injection of FF3 in rats with unilateral hindpaw inflammation on mechanical (PPT (**A**) and PWT (**B**)) and heat (PWL (**C**)) thresholds in inflamed (left panels) and noninflamed (right panels) hindpaws were measured before (0) and 15–60 min after injection, on day 4 after CFA (*P < 0.05, **P < 0.01, ***P < 0.001 vs. vehicle, two-way RM-ANOVA and Bonferroni’s multiple comparison test, n = 9, means ± SEM).

**Figure 3:**
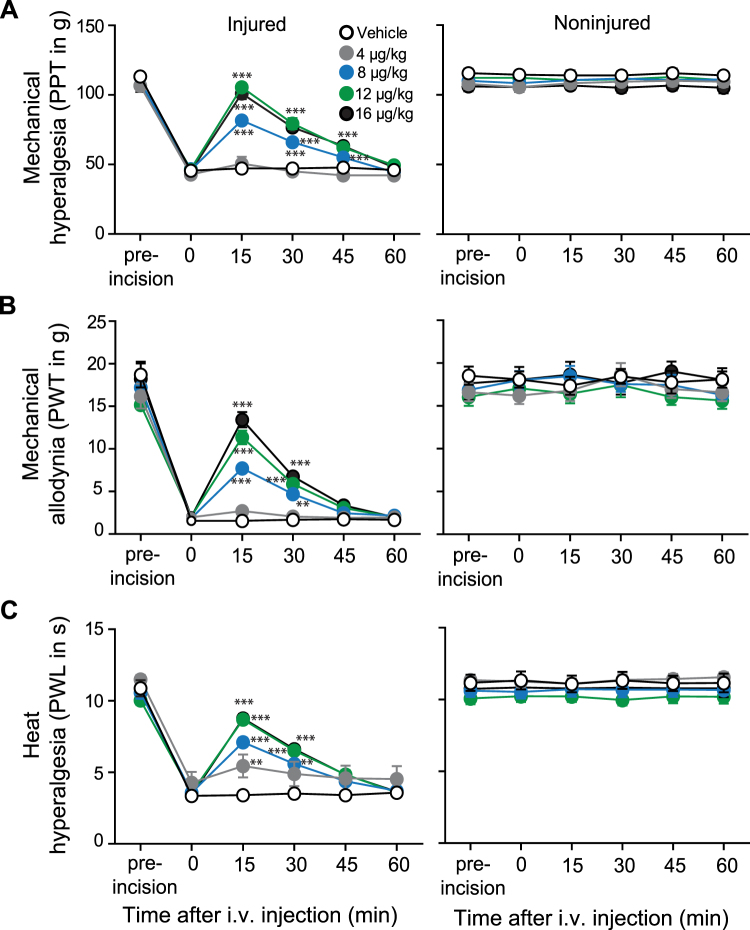
Systemic FF3 dose-dependently reduces incisional pain selectively in injured tissue. Effects after intravenous (i.v.) injection of FF3 in rats with unilateral hindpaw incision. The effects on mechanical (PPT (**A**) and PWT (**B**)) and heat (PWL (**C**)) thresholds in injured (left panels) and noninjured (right panels) hindpaws before (0) and 15–60 min after injection, at 2 h following paw incision (*P < 0.05; **P < 0.01; ***P < 0.001 vs. vehicle, two-way RM-ANOVA and Bonferroni’s multiple comparison test, n = 9, means ± SEM.

**Figure 4:**
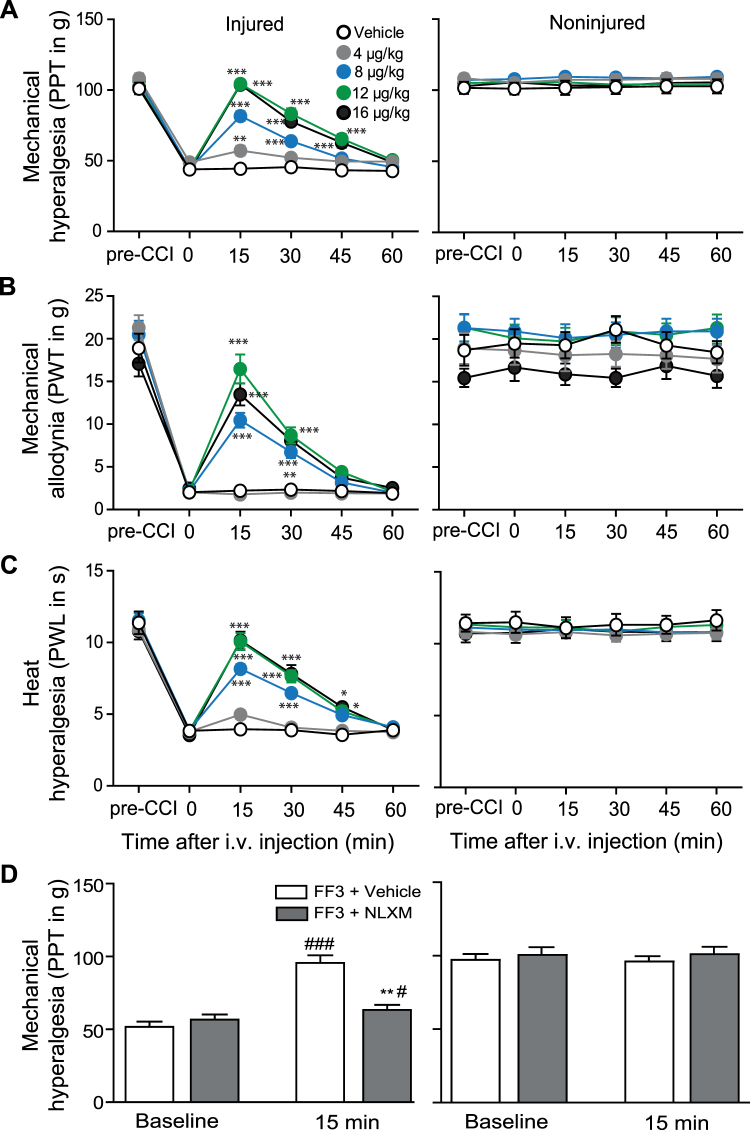
Systemic FF3 dose-dependently reduces CCI-induced pain via peripheral opioid receptors. Effects after intravenous (i.v.) injection of FF3 in rats with CCI on mechanical (PPT (**A**) and PWT (**B**)) and heat (PWL (**C**)) thresholds in injured (left panels) and noninjured (right panels) hindlimbs before (0) and 15–60 min after injection, at 14 days following CCI (*P < 0.05, **P < 0.01, ***P < 0.001 vs. vehicle, two-way RM-ANOVA and Bonferroni’s multiple comparison test, n = 9, means ± SEM). (**D**) Contribution of peripheral opioid receptors to the effects of FF3. NLXM (50 μg) was injected at the site of nerve injury before FF3 (12 μg/kg, i.v.). Effects on PPT were measured 15 min after injection in injured (left panels) and noninjured (right panels) hindlimbs (**P < 0.01 vs. FF3+ vehicle at 15 min, t-test; ^#^*P* < 0.05, ^###^*P* < 0.001 vs. baseline, paired t-test, n = 9, means ± SEM).

To assess analgesic efficacy *in vivo*, we used four clinically relevant rat models of pain, unilateral complete Freund’s adjuvant (CFA)-induced inflammation or plantar incision of the hindpaw, intraperitoneal (i.p.) injection of acetic acid, and unilateral chronic constriction injury (CCI) of the sciatic nerve. Four days following CFA injection (Fig. 2), 2 h after incision (Fig. 3), and 14 days following CCI (Fig. 4), rats developed mechanical hyperalgesia indicated by reduced paw pressure thresholds (PPT), mechanical allodynia manifested by decreased paw withdrawal thresholds (PWT) to von Frey filaments, and heat hyperalgesia indicated by lowered paw withdrawal latencies (PWL) in ipsilateral compared to contralateral paws, and to thresholds before injury.

Intravenous FF3 (4–16 μg/kg, i.v.) produced dose-dependent analgesia manifested by increased PPT, PWT, and PWL between 15–45 min after injection. These effects occurred exclusively ipsilateral to the injuries, without affecting contralateral paws. The most effective doses of FF3 (16 and 12 μg/kg, i.v.) reversed PPT, PWT, and PWL in ipsilateral paws to values before CFA injection (Fig. 2), incision (Fig. 3), or CCI (Fig. 4). Abdominal constrictions (writhings), evaluated over 30 min after i.p. acetic acid (1%), were dose-dependently attenuated by FF3 (4–16 µg/kg, i.v.) injected 5 min after acetic acid (see Supplementary Fig. S1).

To examine the contribution of peripheral opioid receptors, we used naloxone methiodide (NLXM), an opioid receptor antagonist that does not cross the blood-brain barrier^10^, in the CCI model. The analgesic effects produced by FF3 (12 μg/kg, i.v.) in ipsilateral limbs were abolished by NLXM (50 µg) injected at the nerve injury site (Fig. 4D), indicating that FF3-induced analgesia is mediated by local opioid receptors. No changes were observed in contralateral paws (Fig. 4D).

### FF3 induces central and intestinal side effects at high doses

Next, we examined typical opioid side effects mediated centrally or intestinally (sedation, motor impairment, reward, respiratory depression, constipation). Analogous to our previous study^5^, we tested FF3 (30–150 µg/kg) in comparison to the standard opioids fentanyl (30 µg/kg) and morphine (1–64 mg/kg) following subcutaneous (s.c.) injection.

**Figure 5:**
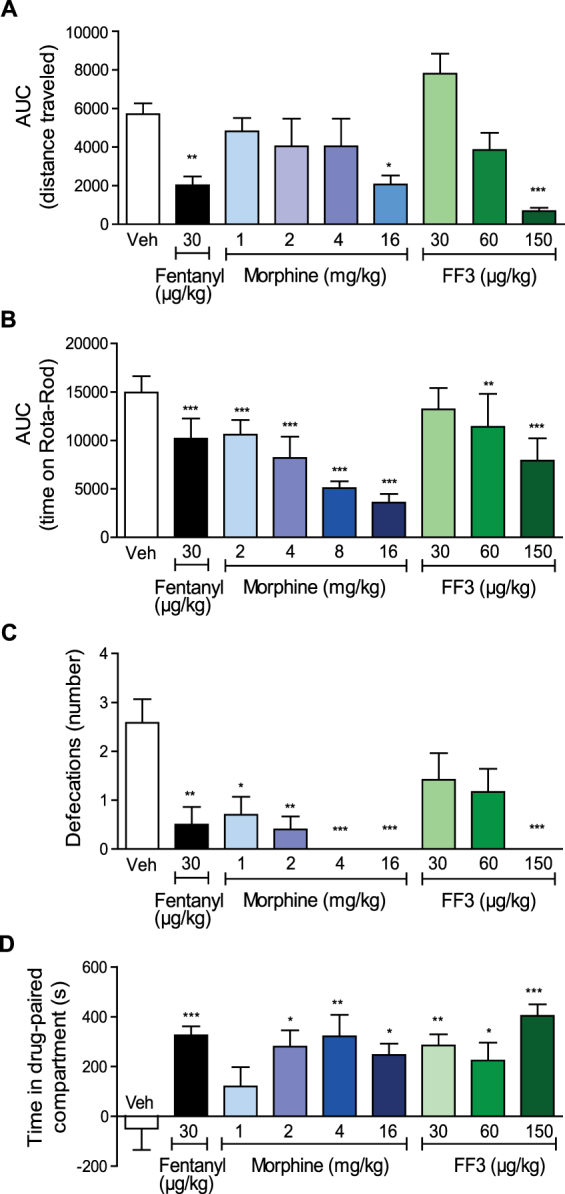
Systemic FF3 induces central and intestinal side effects at high doses. (**A**) Effects of subcutaneous (s.c.) fentanyl, morphine, and FF3 presented as the area under the curve (AUC) of the distance (in cm) travelled during 30 min after drug injection (*P < 0.05, **P < 0.01, ***P < 0.001 vs. vehicle, Kruskal-Wallis one-way ANOVA and Dunn’s multiple comparison test; vehicle, fentanyl and FF3: n = 12, morphine: n = 10, means ± SEM). (**B**) Effects of s.c. fentanyl, morphine, and FF3 presented as the AUC of the time (s) spent on accelerating Rota-Rod at 2, 30 and 60 min after drug injection (**P < 0.01, ***P < 0.001 vs. vehicle, one-way ANOVA and Bonferroni’s multiple comparison test, n = 10). (**C**) Number of defecations in 1 h after s.c. fentanyl, morphine, and FF3 injection (*P < 0.05, **P < 0.01, ***P < 0.001 vs. vehicle, Kruskal-Wallis one-way ANOVA and Dunn’s multiple comparison test; vehicle, fentanyl and FF3: n = 12; morphine: n = 10, means ± SEM). (**D**) Effects of fentanyl, morphine, and FF3 on conditional place preference (*P < 0.05, **P < 0.01, ***P < 0.001 vs. vehicle, one-way ANOVA and Bonferroni’ multiple comparison test; vehicle, fentanyl and FF3: n = 12, morphine: n = 10, means ± SEM).

**Figure 6:**
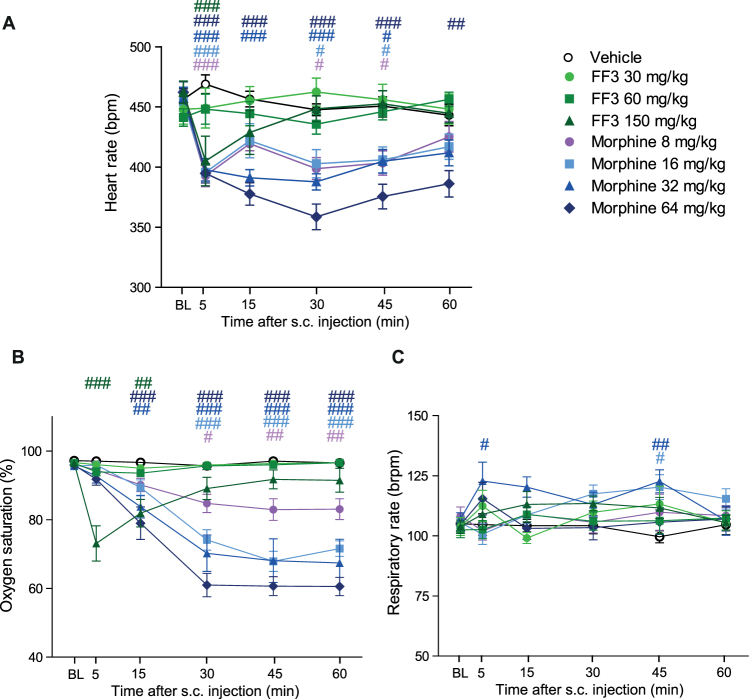
Effects of systemic FF3 on respiration and heart rate. Effects of subcutaneous (s.c.) morphine and FF3 at 5–60 min after injection compared to vehicle on heart rate (beats per min, bpm) (**A**), oxygen saturation (%) (**B**) and respiratory rate (breaths per min, brpm) (**C**) (^#^*P* < 0.05, ^##^*P* < 0.001, ^###^*P* < 0.001 vs. vehicle, two-way RM ANOVA and Bonferroni’s multiple comparison test; vehicle: n = 8; morphine and FF3: n = 10, means ± SEM).

Locomotor activity, measured as the distance traveled within 30 min after drug injections, was decreased by fentanyl (30 µg/kg), and by the highest doses of morphine (16 mg/kg) and FF3 (150 µg/kg) (Fig. 5A). At 5 min after administration of any compound, locomotor activity was significantly lower compared to vehicle (see Supplementary Fig. S2). Impaired motor coordination, measured by an accelerating Rota-Rod, was produced by fentanyl (30 µg/kg), by all doses of morphine (2–16 mg/kg), and by the two highest doses of FF3 (60–150 µg/kg) (Fig. 5B). Constipation, assessed as reduced defecation, was evoked by fentanyl (30 µg/kg), by all doses of morphine (1–16 mg/kg), and by the highest dose of FF3 (150 µg/kg) (Fig. 5C). In the unbiased conditioned place preference test, fentanyl (30 µg/kg), morphine at most doses (2–16 mg/kg), and FF3 at all doses (30–150 µg/kg) produced preference for the drug-associated compartment (Fig. 5D). Heart rate (Fig. 6A) and oxygen saturation (Fig. 6B) were transiently (5–15 min) reduced by FF3 at the highest dose (150 µg/kg), whereas morphine impaired these parameters at all doses (8–64 mg/kg) over the entire period (60 min). Respiratory rate was unaffected by FF3 (30–150 µg/kg), and transiently elevated by morphine (16–32 mg/kg) (Fig. 6C).

## Discussion

We now expanded our concept to develop pain killers that selectively target opioid receptors in peripheral damaged tissues. We have refined the computational simulations to study injury-specific opioid agonists based on decreased pK_a_ values and altered ligand-receptor interactions. Binding and activation of MOR by the new compound FF3 was enhanced at acidic pH. In models mimicking inflammatory, postoperative, neuropathic, and abdominal pain, FF3 dose-dependently reduced nociception exclusively in the injured tissues. Importantly, injury-induced tissue acidosis was measured in inflammatory and postoperative pain models^5^. However, high doses of FF3 induced sedation, motor impairment, reward, constipation, and respiratory depression.

In contrast to other strategies to achieve analgesia without side effects such as ion channel antagonists, bivalent opioid receptor ligands, biased ligands, abuse deterrent opioids (reviewed in^9^), the activation of peripheral opioid receptors selectively eliminates the source of pain generation in injured tissues. To this end, we pursued a novel pharmacodynamics-based approach analyzing interactions between opioid receptors and ligands in injury-induced tissue inflammation and acidosis. These studies underlined the importance of protonation for receptor binding and activation. In contrast to commonly used opioid drugs (Table 2), our previously designed ligand NFEPP with a pK_a_ value reduced to 6.8 displayed enhanced binding and activation of MOR *in vitro*, and analgesic activity selectively in injured tissue without side effects *in vivo*^5^.

**Table 2:** Experimentally determined pK_a_ values of clinically relevant opioid ligands.

Opioid ligand	pK_a_	Reference
Alfentanil	7.82*	^34^
Buprenorphine	8.31	^32^
Butorphanol	8.19	^32^
Codeine	8.21	^32^
Fentanyl	8.43	^32^
Heroin	7.95	^32^
Levorphanol	9.58	^32^
Meperedine	8.59	^32^
Methadone	8.94	^32^
Morphine	8.21	^32^
Nalbuphine	8.71	^32^
Naloxone	7.94	^32^
Oxycodone	8.53	^32^
Oxymorphone	8.17	^32^
Pentazocine	8.88	^32^
Remifentanil	7.51*	^35^
Sufentanil	7.85	^32^
Tramadol	9.41	^32^

^*^No experimental values available, calculated values are stated.

Additional quantum chemical calculations suggested replacement of hydrogens at an alternative position in the fentanyl molecule, as denoted by the derivatives 12 and 13 in Fig. 1A of our previous publication^5^. Among possible electronegative moieties reducing pK_a_ values (in decreasing order of the relative inductive effect: NH_3_^(+)^ > NO_2_ > CN > SO_3_H > CHO > CO > COOH > COCl > CONH_2_ > F > Cl > Br > I > OH > NH_2_ > C_6_H_5_ > H), the fluorine atom is the most suitable because one hydrogen is replaced by only one other atom, thus avoiding significant changes in the overall size or geometry of the molecule. According to our original simulations, the racemic molecule FF3 had an estimated mean pK_a_ value of 5.21 and more negative binding energies (Gibbs free energy, ΔG) under injured/acidic conditions (mean ΔG of H-F12 and H-F13 = −13.45) compared to noninjured/neutral conditions (mean ΔG of H-F12 and H-F13 = −9,25)^5^. Here, we refined our pK_a_ estimation by another method^11,12^. The previous calculations were based on only one three-dimensional structure of each derivative^5^. This is suboptimal, since these molecules are flexible in reality. We now used the structural formula of the molecule as a starting point, and averaged the pK_a_ estimation/calculation for a canonical ensemble of different structures^13^. The recalculated pK_a_ of FF3 (6.01) is now closer to the experimentally obtained value (7.22). However, there are still differences between calculated (both methods) and experimentally determined pK_a_ values, which are most likely due to the difficulty to determine solvation energies by computational methods^14^. Although generally recommended^12^, neither of these calculations used experimentally obtained pK_a_ values of related molecules because such measurements were not available so far. Previous publications on computational methods for pK_a_ estimation suggest that approaches based on first principles (as in the present study) are preferable to empirical models based on statistics^15^. Therefore, a combination of our method with experimentally obtained pK_a_ values of related molecules appears most promising for the future.

Some results obtained with FF3 were similar to NFEPP^5^: FF3-induced MOR binding and G-protein dissociation dropped at physiological pH compared to acidic conditions (Table 1). Mechanical and thermal hyperalgesia as well as mechanical allodynia were reduced exclusively in injured limbs without effects in the contralateral uninjured limbs. Additionally, FF3 produced dose-dependent analgesia in the abdominal writhing assay. NLXM reversed the analgesic effect of FF3 in the CCI model, supporting the selective activation of peripheral opioid receptors. In contrast to NFEPP, FF3 produced side effects at higher dosages than those eliciting analgesia. In particular, our finding that FF3 induced conditioned place preference indicates its abuse liability. It is most likely that a pK_a_ value of 7.22 is not low enough to restrict FF3′s activity to injured/acidic tissue, although analgesic doses did not affect contralateral/healthy limbs in the nociceptive tests. This supports our hypothesis that the pK_a_ of a ligand should be close to the pH of injured tissue (as shown for NFEPP, see Table 1) to obtain selective peripheral analgesia without side effects. In addition, possible changes of receptor conformation due to acidosis-induced altered protonation states of amino acid residues must be taken into account in the design of ligands. Thus, further refinement of the computational calculations is necessary to enable reliable pK_a_ predictions leading to novel structures of peripherally selective agonists.

## Methods

### Quantum chemical pK_a_ calculations

The pK_a_-values were estimated using Gaussian 09^16^ and the quantum-chemical method published previously^13^. To predict Gibbs free energy changes correlated with deprotonation in water, the geometries of fentanyl and FF3, in gas-phase and in solution, were fully optimized employing density functional theory using B3LYP with the 6–31 + G* basis set (Becke’s three-parameter hybrid exchange functional and Lee-Yang-Parr correlation functional). In solvation calculations, the integral equation formulation of the polarizable continuum model (IEF-PCM) was applied. Thermal corrections to the Gibbs free energy at 298.15 K were determined at the same level. To consider the dependency of the pK_a_-value on the molecular conformation^13^, pK_a_ calculations for either molecular species were performed from 10 different starting conformations, generated by 10 ns gas-phase molecular dynamics simulations at 298.15 K using GROMACS 2016.1^17^.

### Chemicals/Drugs

Fentanyl citrate, naloxone hydrochloride (NLX), NLXM, Guanosine 5′- [γ-thio]triphosphate tetralithium salt (cold GTPγS) and Guanosine 5′-diphosphate sodium salt (GDP) were purchased from Sigma-Aldrich (Taufkirchen, Germany). [^3^H]-DAMGO and [^35^S]-GTPγS were purchased from Perkin Elmer (Rodgau-Jügesheim, Germany).

FF3 was synthesized according to our design by a contractor (ASCA GmbH, Berlin, Germany) leading to the racemate FF3 (Fig. 1B). The experimental measurement of the pK_a_ value was performed by a contractor (Sirius Analytical Ltd., Forest Row, UK). For *in vitro* experiments, fentanyl and FF3 were initially dissolved in water or dimethyl-sulfoxide (DMSO) and diluted in assay buffer to final concentrations. For *in vivo* experiments, FF3 was dissolved in DMSO and diluted with 0.9% NaCl to obtain the final concentrations. The maximum DMSO concentration was 4.2% for s.c., and 0.5% for i.v. injections. Fentanyl and NLXM were dissolved in 0.9% NaCl. Control groups were treated with DMSO or NaCl, respectively.

### Cell cultures

Wild type and MOR-expressing HEK293 cells were maintained in DMEM media (Sigma-Aldrich, Taufkirchen, Germany) supplemented with 10% fetal bovine serum and 1% penicillin/streptomycin in the absence or presence of 0.1 mg/ml geneticin (Biochrom AG, Berlin, Germany), respectively, in 5% CO_2_ at 37 °C. According to their confluence, cells were passaged 1:3–1:10 every second to third day from P8 to P28^18^.

### Radioligand binding assays

HEK293 cells stably expressing rat MOR were cultured in flasks with a growth area of 175 cm². Cells were washed twice with ice-cold assay buffer (Trizma® Preset Crystals, 50 mM, pH 7.4) (Sigma-Aldrich), then scratched from the culture flask in 10 ml ice cold assay buffer, homogenized and centrifuged twice at 42,000 *g* for 20 min at 4 °C as described previously^7,19,20^. Protein concentration was determined according to the Bradford method^21^. The half maximal inhibitory concentration (IC_50_) of FF3 required to displace 4 nM of the standard MOR ligand [^3^H]-DAMGO from the receptor was determined at pH values 6.5 and 7.4. A protein amount of 80–100 µg was incubated with 4 nM [^3^H]-DAMGO (50 Ci/mmol) and FF3 dissolved in 50 mM assay buffer at pH 6.5 or 7.4 for 90 min at room temperature. Nonspecific binding was determined by the addition of 10 µM NLX^20^. Filters were soaked in 0.1% polyethyleneimine solution before use. Bound and free ligands were separated by rapid filtration under vacuum through Whatman GF/B glass fiber filters. Bound radioactivity was determined by liquid scintillation spectrophotometry at 69% counting efficiency for [^3^H] after overnight extraction of the filters in scintillation fluid. For [^35^S]-GTPγS-binding experiments, membranes were prepared as described above. After determination of protein concentration, membranes were centrifuged as described above and resuspended in [^35^S]-GTPγS-binding assay buffer (100 mM NaCl, 50 mM Tris Base, 5 mM MgCl_2_, 0.1 mM EGTA, 0.2% bovine serum albumin, 10 mM dithiotreitol and 0.03 mM GDP) adjusted to pH 7.4 or 6.5^22^. A protein amount of 50 μg was incubated with 0.05 nM [^35^S]-GTPγS and varying concentrations of fentanyl or FF3 at the respective pH for 2 h at 30 °C to determine dose response curves and EC_50_ values. Whatman GF/B glass fiber filters were soaked in water before use. Bound and free [^35^S]-GTPγS was separated *via* rapid filtration as described above but for [^35^S]. Nonspecific binding was determined by the addition of 10 μM cold GTPγS. Basal [^35^S]-GTPγS-binding was measured in the absence of opioid ligand and cold GTPγS.

### Transfection for FRET measurements

Culture and transfection of wild type HEK293 cells were performed as previously described^5^. Briefly, cells were grown for 24 h (70–90% confluence) on 24 mm diameter glass coverslips coated with poly-L-lysine before transfection. 0.3–1 μg cDNA per construct were transfected with X-treme GENE HP DNA reagent (Sigma-Aldrich, Taufkirchen, Germany) following the supplier’s recommendations. The plasmid containing the cDNA encoding the FLAG-epitope-tagged rat MOR (oprm1, NM_013071.2) in pcDNA™3.1 vector was provided by Prof. C. Zöllner (University Hamburg, Germany). Rat Gα_i1_ (Gnai1, NM_013145.1) was tagged within the αb-αc loop (between residues 121 and 122) with a truncated enhanced cyan fluorescent monomeric Turquoise (mTqΔ6), to force the fluorophore into a FRET-promoting orientation^23^. Gα_i1_-mTqΔ6 and tetracysteine (TC)-tagged-Gβ_1_ (Gnb1, NM_030987.2) were subcloned into pcDNA™3.1 expression vector between BamH1 or EcoRI and XhoI, respectively. A human construct with circular permutated (cp) Venus-Gγ_2_ was kindly provided by Dr. M. Adjobo-Hermans (Department of Biochemistry, Radboud Institute for Molecular Life Science, The Netherlands). The rat variant of cpVenus-Gγ_2_ (Gng2, NM_001257349.1) and the PTX-resistant Gα_i1_-mTqΔ6 were generated by mutagenesis (N24S and C351I, respectively) using QuikChange™ Mutagenesis kit, and then checked by sequencing (Source Bioscience Sequencing, Berlin, Germany). The functionality of the transfected G-protein subunits was confirmed by preserved cAMP inhibition by fentanyl in the presence of PTX.

### Real-time FRET measurements

At 48 h after transfection of HEK293 cells with MOR, Gβ_1_, Gα_i1_-mTqΔ6, and cpVenus-Gγ_2,_ FRET between the latter two proteins was measured. Cells were briefly washed with PBS and subsequently equilibrated with assay buffer (140 mM NaCl, 5 mM KCl,2 mM CaCl_2_, 2 mM MgCl_2_, 10 mM HEPES, 10 mM Glucose) at different pH values. We used a FRET Nikon Eclipse TE2000-S widefield microscope equipped with a ultra-high-quality monochrome camera DS-Qi1, a Polychrome V monochromator and a DualView beam splitter to separate yellow fluorescent protein (YFP) (535 ± 15 nm) and cyan fluorescent protein (CFP) (480 ± 20 nm) emissions. Images of donor-donor (DD; excitation 439 nm/emission 480 nm), donor-acceptor (DA; excitation 439 nm/emission 535 nm) and acceptor-acceptor (AA; excitation 505 nm/emission 535 nm) were taken with a 40X objective (S fluor/1.30 oil) every 1 s with exposure times kept constant and comprised between 50 (for Venus) and 150 ms (for mTq). Fluorescence intensities were quantified on regions of interest (ROI) including the whole cell by NIS-Elements software. After background subtraction, NIS-Elements calculated FRET efficiency (in %), using the correction coefficients (CoA = 0.19; CoB = 0.43), accounting for bleed-through and direct YFP excitation from 3 independent experiments. On the day of the experiment, cells were washed and incubated with assay buffer (at pH 7.4 or 6.5) and after 20 s of live imaging, fentanyl, FF3 or vehicle (0.1% DMSO) were added at various concentrations for the remaining time. The experimenter was blinded to the treatment and buffer pH. ROIs with measured fluorescence intensities lower than 100 RFU in any of the recorded channels (DD, DA or AA) and with FRET % values lower than 1 were excluded prospectively. These exclusion criteria were established in preliminary experiments (showing unstable traces over time for ROIs corresponding to these exclusion criteria) and applied before unblinding. For each ROI, background-corrected fluorescence intensities (RFU ≥ 100) and corresponding FRET parameters (FRET efficiency ≥ 1%) over time were normalized to initial values (=1), pooled by experiment and finally averaged across the experiments. ΔFRET values for each treatment were derived by subtracting initial from final normalized FRET %. Finally, vehicle ΔFRET % was subtracted from each treatment in a pH-paired manner to yield net ΔFRET %.

### Animals

Experiments were performed in male Wistar rats (200–300 g, Janvier, France) and were approved by the State animal care committee (Landesamt für Gesundheit und Soziales, Berlin). All experiments were performed in accordance with the relevant guidelines and regulations. The sample size was calculated with the G*Power 3.1.2 program (80% power and 0.05 level of significance). Animal experiments were stopped when specific termination criteria were reached (approved by the State animal care committee). The experimenter who performed experiments and analyzed the results was blinded to the interventions. Rats were kept on a 12 h dark-light cycle in groups of 3 in cages lined with ground corncob bedding with free access to food and water *ad libitum* and constant room temperature and humidity of 22 ± 0.5 °C and 60–65%, respectively. Before nociceptive testing, handling was performed once per day for 1–2 min. Animals were habituated to the test cages once or twice a day for 15 min, starting 4 days prior to the experiments. Statistical power calculations were performed to obtain the minimal number of animals for the experiments. After termination of the experiments, rats were killed by an overdose of isoflurane (AbbVie, Ludwigshafen, Germany).

### Paw inflammation

Rats received an i.pl. injection of CFA (150 µl; Calbiochem, La Jolla, CA, USA) into the right hindpaw under brief isoflurane anesthesia^24^. Nociceptive testing was performed 4 days after CFA injection.

### Paw incision

Unilateral hindpaw incision was induced under isoflurane anesthesia^25^. A 1 cm longitudinal incision through skin and fascia of the plantar aspect of the paw was made with a No. 11 blade. The starting point was 0.5 cm proximal of the heel’s edge and extending toward the toes. The plantaris muscle was lifted and incised longitudinally. The muscle origin and insertion remained intact. The wound was closed with silk sutures and experiments were performed 2 h after incision.

### Abdominal writhing

Animals received an i.p. injection of 10 ml/kg 1% acetic acid and were placed individually into transparent cages for observation of abdominal constrictions (“writhing”)^26^. Writhes were recorded 5–35 min thereafter.

### Nerve injury

Chronic constriction injury (CCI) of the sciatic nerve was induced under isoflurane anesthesia^27^. At the level of the left mid-thigh, 4 loose 4/0 silk ligatures were placed around the nerve and the wound was closed with silk sutures. Experiments were performed 14 days after CCI.

### Injections and experimental protocols

Brief isoflurane anesthesia was applied for i.v. (200 μl), i.p., and injections at the site of the nerve injury (100 μl). Subcutaneous (s.c.) injections were performed without anesthesia. Nociceptive tests were performed in separate groups of animals, before and 5–60 min after injections. NLXM was injected at the site of nerve injury immediately before i.v. injection of agonists. Pain thresholds were measured 15 min after injections. All dosages were determined in pilot experiments. The experimenter was blinded to the doses and drug treatments.

### Mechanical hyperalgesia (Randall-Selitto test)

Rats were gently held under paper wadding and incremental pressure was applied via a wedge-shaped, blunt piston onto the dorsal surface of the hindpaws using an automated gauge (Ugo Basile, Comerio, Italy). The PPT necessary to induce paw withdrawal was determined by averaging three consecutive trials separated by 15 s intervals. The cut-off was set at 250 g to avoid tissue damage. The sequence of paws was alternated between animals to preclude order effects.

### Mechanical allodynia (von Frey test)

Rats were placed separately in clear Plexiglas cubicles located on a stand with anodized mesh. PWT was measured as described elsewhere^28^. Briefly, the plantar surface of the hindpaw was stimulated with von Frey filaments with increasing force. PWT was reached if a filament produced withdrawal responses to 3 stimuli. The strength of calibrated von Frey filaments was 0.6, 1, 1.4, 2, 4, 6, 8, 10, 15 and 26 g.

### Heat hyperalgesia (Hargreaves test)

Rats were placed separately in clear Plexiglas cubicles located on a stand with a glass surface. A high-intensity light bulb generated radiant heat was applied to the plantar surface of the hindpaws from underneath the glass surface. PWL was measured using an electronic timer as described^29^. Three measurements separated by at least 10 s were averaged. The heat intensity was adjusted to obtain a baseline withdrawal latency of about 10–12 s in uninjured paws, and the cut-off was 20 s.

### Conditioned Place Preference (CPP)

We used an unbiased counterbalanced CPP protocol in healthy rats as described previously^5,30,31^. The CPP boxes consist of light- and sound-attenuating chambers (60 × 30 × 30 cm; Ugo Basile) with two compartments separated by a removable door. The two compartments differed in wall color (black or white with black stripes) and floor texture (“grid” or “hole”). For habituation, each rat was placed into the chamber without separator to freely explore it for 30 min (days 1 and 2). In the pre-conditioning phase, each rat was placed into the unseparated chamber for 15 min and the time spent in each compartment was recorded (day 3). Rats showing high unconditioned preference for one of the two compartments were excluded from further analysis, resulting in slightly differing animal numbers (n = 10–12). During conditioning, treatment and treatment-associated compartments were assigned randomly. Each rat underwent three 60 min conditioning sessions receiving the test substance s.c. in one compartment (one session every other day), and three 60 min sessions receiving vehicle in the other compartment on the alternate days (days 4–9). On the test day (day 10), rats did not receive any drug and were allowed to freely explore the entire chamber (without separator) for 15 min. The time spent in each compartment was recorded using the AnyMaze software (Ugo Basile). Place preference was calculated as time spent in the drug-associated compartment subtracted by the time spent in the vehicle compartment.

### Locomotor activity

Horizontal locomotor activity of healthy rats was measured in the CPP apparatus on the first day of conditioning of the CPP protocol (day 4). Locomotion was recorded by an infrared camera coupled to a computer with AnyMaze software (Ugo Basile) and was measured as the distance (in cm) traveled in 5 min intervals during 30 min after s.c. drug administration.

### Motor coordination (Rota-Rod test)

On the first day naïve rats were trained on the Rota-Rod (Ugo Basile) at 5 rotations per min (rpm). On days two and three the rotating speed was increased to 10 rpm until rats were able to stay on the Rota-Rod for 300 s (maximum 5 trials). On the testing day the data were recorded for three baseline trials prior to drug exposure, followed by three trials at 2, 30 and 60 min after s.c. drug injection at an accelerating speed (10–60 rpm over 300 s). The latency to fall was recorded for 3 successive attempts and averaged for each trial.

### Respiratory depression and heart rate

We used a pulse oximeter (MouseOx, StarLife Sciences, Pittsburgh PA, USA) to measure heart rate, respiratory rate, and blood O_2_ saturation, as described previously^5^. Briefly, after 2 days of handling and 2 days of habituation (30 min/session), healthy rats were placed in a Plexiglass box wearing the pulse oximeter clip around the neck. The mean of two representative, error-free data sets, were calculated at each time point after s.c. injections.

### Defecation

Excreta of individual rats were collected and counted for 1 h after s.c. drug administration on the first conditioning day in the CPP boxes.

### Data handling and statistical analyses

All data were assessed for normal distribution and equal variances by Kolmogorov-Smirnov test and/or D’Agostino test, and Pearson test. In dose-response experiments (displacement binding and GTPγS-assay), means of values at each agonist concentration and each pH were calculated and used to derive IC_50_ and EC_50_ by nonlinear regression and were then subjected to unpaired t-test. In FRET experiments, dose-dependent responses were assessed by nonlinear regression setting as a constraint the maximal effect for each drug/pH condition. Normally distributed data were analyzed by one-way repeated measurements (RM)-ANOVA followed by Dunnett’s test.

Behavioral data were expressed as raw values or area under the curve. Two-sample comparisons were made using paired or unpaired t-test for normally distributed data, or Wilcoxon or Mann-Whitney test for non-normally distributed data. Changes over time (more than two time points) after one treatment were evaluated using one-way RM-ANOVA followed by Bonferroni test for normally distributed data, or Friedman one-way RM-ANOVA followed by Dunn’s test for non-normally distributed data. Two-way RM-ANOVA and Bonferroni test were used to compare two parameters over time. Multiple comparisons at one time point were performed using one-way ANOVA followed by Dunnet’s test or Bonferroni’s multiple comparison test for normally distributed data, or by Kruskal-Wallis one-way ANOVA followed by Dunn’s test for non-normally distributed data. Differences were considered significant if *P* < 0.05. Prism 5 (GraphPad, San Diego, USA) was used for all tests and graphs and all data were expressed as means ± standard error of the mean (SEM).

## Electronic supplementary material

Supplementary information
