# Supplementary material for: Opioid receptor signaling, analgesic and side effects induced by a computationally designed pH-dependent agonist
Source: Sci Rep. 2018 Jun 12;8:8965. doi: 10.1038/s41598-018-27313-4 (PMC5997768; doi:10.1038/s41598-018-27313-4)
Supplement: Supplementary file 1 — Supplementary information [file 41598_2018_27313_MOESM1_ESM.pdf]

Supplementary information for

Opioid receptor signaling, analgesic and side effects induced by a computationally designed pH-dependent agonist

Viola Spahn[1], Giovanna Del Vecchio[1], Antonio Rodriguez-Gaztelumendi[1,†], Julia Temp[1], Dominika Labuz[1], Michael Kloner[1], Marco Reidelbach[2], Halina Machelska[1], Marcus Weber[3], Christoph Stein[1]*

*To whom correspondence should be addressed: E-mail: Christoph.stein@charite.de

Supplementary Materials:

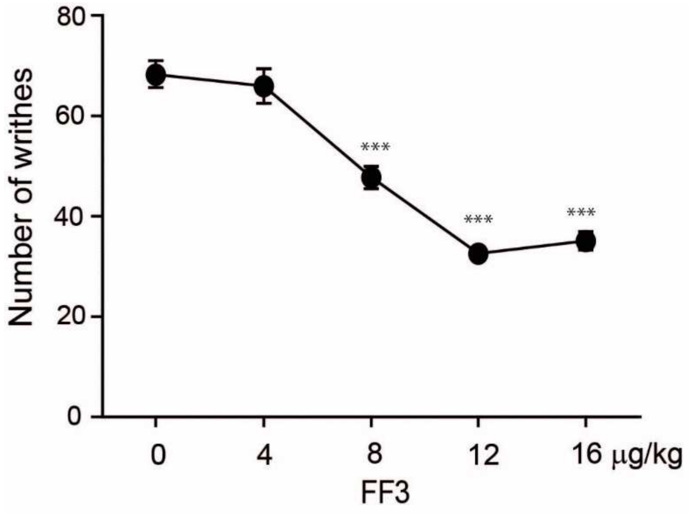

Supplementary Fig. S1. *Systemic FF3 dose-dependently reduces abdominal writhing.* Effects after intravenous injection of FF3 on number of writhes during 5-35 min after intraperitoneal 1 % acetic acid injection (*** $P < 0.001$ vs. “0”, one-way ANOVA and Bonferroni’s multiple comparison test, n = 9 rats per group, means ± SEM).

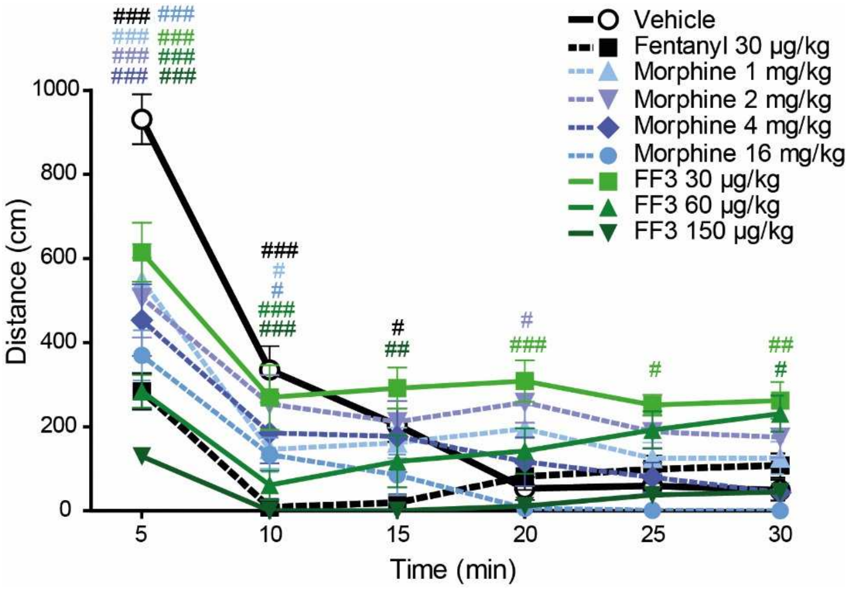

Supplementary Fig. S2. *Systemic FF3 induces sedation at high doses.* Effects of subcutaneous fentanyl, morphine, and FF3 on locomotor activity presented as the distance (in cm) travelled during 30 min after drug injection (# $P < 0.05$, ## $P < 0.01$, ### $P < 0.001$ vs. vehicle, two-way RM-ANOVA and Bonferroni's multiple comparison test; vehicle, fentanyl and FF3, n=12, morphine, n=10, means ± SEM).

Supplementary text

FF3, morphine, and fentanyl induced significantly decreased locomotor activity at 5 min after injection compared to vehicle. This effect was not seen for morphine (2 mg/kg) or FF3 (30 and 60 µg/kg) at later time points. At time points 20, 25, and 30 min FF3 (30 µg/kg) and at 30 min (60 µg/kg), locomotor activity was significantly increased compared to vehicle.
